# Two Possible Strategies for Drug Modification of Gemcitabine and Future Contributions to Personalized Medicine

**DOI:** 10.3390/molecules27010291

**Published:** 2022-01-04

**Authors:** Mariana Pereira, Nuno Vale

**Affiliations:** 1 OncoPharma Research Group, Center for Health Technology and Services Research (CINTESIS), Rua Doutor Plácido da Costa, 4200-450 Porto, Portugal; mariana.m.pereira2097@gmail.com; 2 Department of Community Medicine, Health Information and Decision (MEDCIDS), Faculty of Medicine, University of Porto, Alameda Professor Hernâni Monteiro, 4200-319 Porto, Portugal

**Keywords:** gemcitabine, drug repurposing, cell-penetrating peptides, PBPK

## Abstract

Drug repurposing is an emerging strategy, which uses already approved drugs for new medical indications. One such drug is gemcitabine, an anticancer drug that only works at high doses since a portion is deactivated in the serum, which causes toxicity. In this review, two methods were discussed that could improve the anticancer effect of gemcitabine. The first is a chemical modification by conjugation with cell-penetrating peptides, namely penetratin, pVEC, and different kinds of CPP6, which mostly all showed an increased anticancer effect. The other method is combining gemcitabine with repurposed drugs, namely itraconazole, which also showed great cancer cell inhibition growth. Besides these two strategies, physiologically based pharmacokinetic models (PBPK models) are also the key for predicting drug distribution based on physiological data, which is very important for personalized medicine, so that the correct drug and dosage regimen can be administered according to each patient’s physiology. Taking all of this into consideration, it is believed that gemcitabine can be repurposed to have better anticancer effects.

## 1. Modification and Repurposing Drugs in Cancer

Drug development needs a program that offers comprehensive drug conjugation strategies that can be designed and developed to fit prolonged pharmacological activity, allowing the reduction of the frequency of drug administration and thus improving the patient’s quality of life. One way to reduce risk and maintain drug levels involves drug repurposing. Today, a wide variety of anticancer drugs are approved and are being used in the clinic for patient treatment. However, there is a lack of effectiveness and positive outcomes in many cancer patients after the use of these drugs. This has incited a continuous hunt for new anticancer drug candidates, but these require high investments from pharmaceutical industries and long periods to develop. Recently, drug repurposing has emerged as a strategy to reduce development times and cancer treatment costs, speeding up access to new cancer therapies [1,2]. Drug repurposing is a drug development strategy that aims to reuse already existing licensed and well-characterized drugs for new medical indications. In this context, research focuses on finding untapped anticancer potential in non-anticancer drugs, which may result in new clinical applications in cancer therapy. This way, even if it requires a new dosage regimen assessment, one can rely on previous pharmacokinetic, toxicological, and safety data obtained from years of widespread clinical use [3,4].

Amongst the wide range of non-cancer drugs currently being studied for integration into cancer treatments are itraconazole and verapamil, with at least one peer-reviewed paper supporting their use against cancer [5,6,7]. Both have shown a survival benefit in at least one randomized trial. For example, itraconazole has shown survival benefit against metastatic non-squamous non-small-cell lung cancer while verapamil has been reported to increase the survival of patients with anthracycline-resistant metastatic breast carcinoma [5,7]. Despite both showing promise, itraconazole has been proven to be the more relevant out of the two drugs for cancer treatment, with more successful cases and applications in a wider range of cancer types [8,9]. Verapamil clinical evidence of anticancer activity enhancement has been revealed, on the other hand, to be more limited [10,11].

Another promising drug that could be altered (by chemical modification or combination with other drugs) to have an increased anticancer effect is gemcitabine, which will be the focus of this review. Physiologically based pharmacokinetic (PBPK) models and how they can help in drug repurposing will also be discussed.

## 2. Gemcitabine

Gemcitabine is a nucleoside-analog chemotherapeutic drug that acts against an extensive range of solid tumors, such as pancreatic, breast, ovarian, and non-small-cell lung cancers [12]. This anticancer drug has several mechanisms of action. One is the inhibition of thymidylate synthetase, which is important for the inhibition of DNA synthesis and cell death. Another one is the activation of the c-Jun *N*-terminal kinase (JNK) and the p38 mitogen-activated protein kinase (p38 MAPK). These are key kinases in two major stress-activated signaling pathways [13].

**Figure 1:**
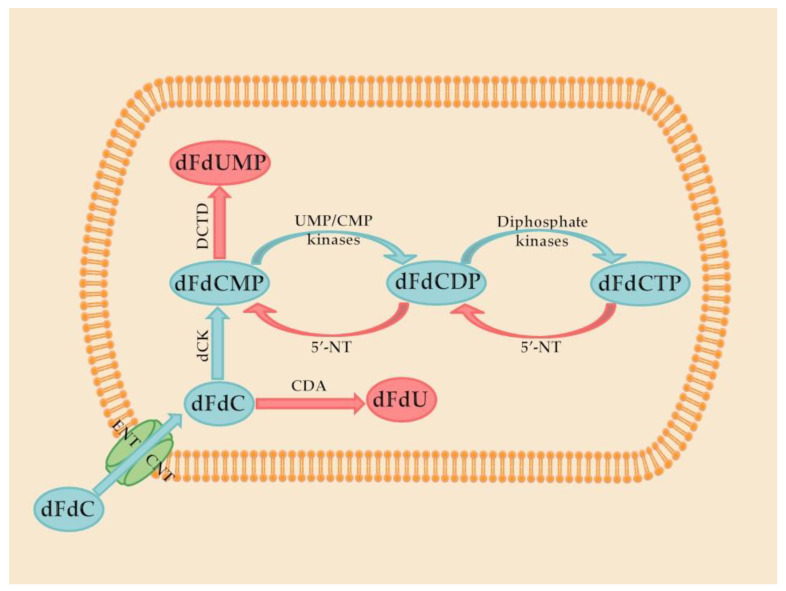
Phosphorylation and dephosphorylation of gemcitabine (dFdC) in the cell. ENT: equilibrative nucleoside transporters, CNT: concentrative nucleoside transporters, dFdCMP: gemcitabine monophosphate, dFdCDP: gemcitabine diphosphate, dFdCTP: gemcitabine triphosphate, dFdU: 2′,2″-difluorodeoxyuridine, dFdUMP: 2′,2′-difluorodeoxyuridine monophosphate, CDA: cytidine deaminase, DCTD: deoxycytidylate deaminase, 5′-NT: 5′-nucleotidase, UMP/CMP kinase: nucleoside monophosphate kinase. Upper left corner is the gemcitabine molecular structure.

Gemcitabine is a prodrug that activates after intracellular conversion to two active metabolites, gemcitabine diphosphate and gemcitabine triphosphate, by the enzyme deoxycytidine kinase. Gemcitabine diphosphate inhibits ribonucleotide reductase, the enzyme responsible for catalyzing the synthesis of deoxynucleoside triphosphates, required for DNA synthesis. Gemcitabine triphosphate (diflurorodeoxycytidine triphosphate) competes with endogenous deoxynucleoside triphosphates for incorporation into DNA [12]. These steps are demonstrated in Figure 1.

However, treatment with gemcitabine has limited efficacy due to its high toxicity and inactivation in the serum, through deamination of its *N*-4 amine. Another disadvantage associated with this drug is that, after initial tumor regression, some tumor cells may develop different forms of drug resistance, such as resistance related to nucleoside transporter deficiency. Indeed, the expression of human equilibrative nucleoside transporter-1 (hENT-1), which plays a key role in gemcitabine intracellular uptake, was found to decrease in resistant cell lines [14].

**Figure 2:**
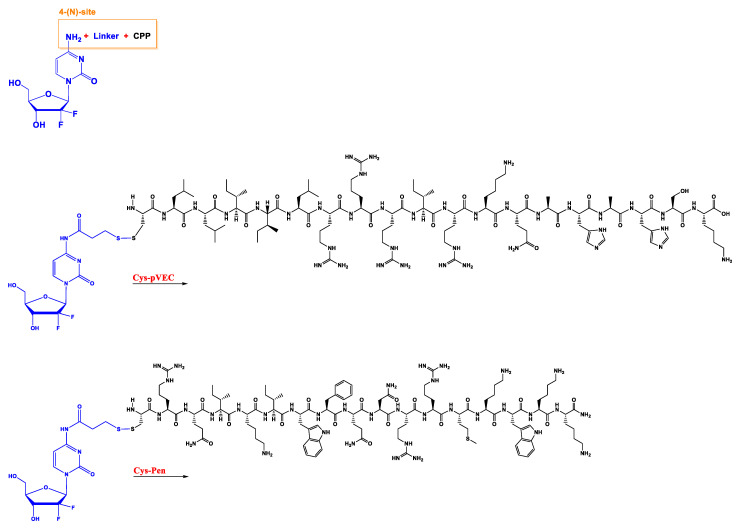

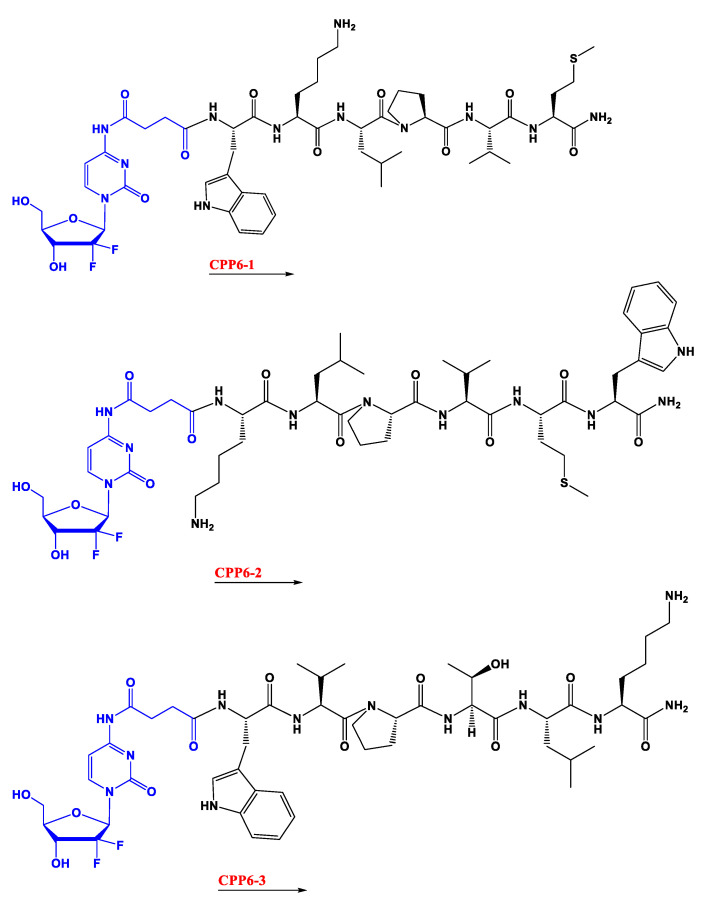
Principal modification site in gemcitabine molecule and different cell-penetrating peptides (Cys-pVEC, Cys-Pen, CPP6-1, CPP6-2, and CPP6-3) used in preparation on new conjugates of this drug.

To improve gemcitabine metabolic stability and cytotoxic activity, as well as to limit tumor drug resistance, several alternatives have emerged, such as the synthesis of prodrugs [12,15,16,17]. Indeed, chemical modifications could, potentially, lead to new therapeutic strategies. Various modifications have already been performed in the 4-(*N*)- and 5′-positions of gemcitabine (Figure 2), such as the incorporation of poly(ethylene glycol) (PEG), valproic acid, 1,1′,2-tris-nor-squalenoic acid (squalene) or valeroyl, heptanoyl, lauroyl, and stearoyl linear acyl derivates in the 4-(*N*) site, and the addition of fatty acid chains or phosphate-function-protecting groups to the 5′-position [12].

Additionally, in a recent work developed in our research group, hexapeptide was conjugated to a 4-(*N*)- site through an anhydride succinic linker (Figure 2). Other than chemical modification, a strategy commonly used in cancer treatment to overcome drug resistance and improve efficiency is drug combination [18]. These two strategies will be discussed in the following sections, with examples of works performed by our group to support chemical alteration or drug repurposing of gemcitabine.

## 3. Peptide-Gemcitabine Conjugates

As mentioned previously, the chemical modification of gemcitabine to increase anticancer effects is growing in interest. One of the groups with most potential to be conjugated with gemcitabine is the cell-penetrating peptides (CPPs), and our group has two types of CPPs that demonstrate this potential [19,20].

In the first study, the aniline moiety of gemcitabine was conjugated with two CPPs to ease intracellular entrance. The CPPs used were penetratin (Pen) and pVEC (LLIILRRRIRKQAHAHSK) coupled with cysteine (Cys) residue on the *N*-terminus of both, originating the final CPPs, cysteine-penetratin-coupled CPP (Cys-Pen) and cysteine-pVEC-coupled CPP (Cys-pVEC). These were conjugated with a gemcitabine derivate (modified with a thiopyridyl group) through a 3-sulfanylpropanoyl linker. Through a semi-quantitative study, it was demonstrated that gemcitabine is released after hydrolytic cleavage of both conjugates, with 9.6 day and 42 h half-lives for gemcitabine conjugated with Cys-Pen CPP (Cys-Pen-Gem) and gemcitabine conjugated with Cys-pVEC CPP (Cys-pVEC-Gem), respectively [19].

After this, the conjugates’ antiproliferative activity was tested in gastric (MKN-28), colon (HT-29), and colorectal adenocarcinoma (Caco-2) cancer cells using the protein-binding dye sulforhodamine B technique. Almost all conjugates had a higher effect than gemcitabine or the CPPs alone in all cells, except for Cys-pVEC-Gem on Caco-2 cells. This may be because that conjugate has lower relative hydrolytic stability, and, therefore, lower internalization by Caco-2 cells. On the other hand, this conjugate had a higher effect on MKN-28 cells, in which the lower stability and consequent rapid increase in gemcitabine concentrations might allow the drug to have a higher antiproliferative impact before its inactivation. In HT-29 cells, a concentration-dependent effect was determined for the conjugates [19].

These results revealed that these CPPs have promise in boosting gemcitabine antiproliferative activity, although more conjugates, tests, and variability of cells are needed to fully confirm this hypothesis [19].

Further study of these two conjugates was performed by using in silico methods, namely GastroPlus^TM^ (amongst others) to evaluate their pharmacokinetics and understand how their physicochemical properties affect their penetration capacity [21]. The results showed that the conjugate Cys-pVEC-Gem had the best profile for drug delivery. It binds less with plasma proteins (probably due to low distribution volume), which leaves a higher percentage of the compound free in the blood, allowing it to reach the targets intended. It also showed the best bioactivity, with fast release and high max concentration (C_max_). When accounting for the pharmacokinetic potential, the properties calculated in silico, and the in vitro bioactivity, Cys-pVEC-Gem conjugate is the most promising for future therapeutic use and is the better option for more study [21].

In the second study, gemcitabine was instead conjugated with three kinds of cell-penetrating hexapeptides (CPP6). These were obtained by adding the amino acid tryptophan (Trp) to two cell-penetrating pentapeptides (CPP5), a Bax inhibitor peptide V5 (VPMLK), and a mutant of Bax inhibitor peptides that lacks inhibitory activity (KLPVM), to facilitate cell penetration. The addition was in the *N*-terminal (CPP6-1) of KLPVM and the C-terminal and *N*-terminal of the VPMLK (CPP6-2 and CPP6-3, respectively). A derivate of gemcitabine (with two hydroxyl groups) was firstly conjugated with a succinic anhydride linker (that enhances drug delivery rate) and then with each of the CPP6. Tests to determine inhibition of cell growth of the conjugates and reference drugs (tamoxifen and metformin) were performed in the human prostate (PC-3), human pancreatic (BxPC-3), and human breast (MCF-7) adenocarcinoma cell lines, through sulforhodamine B dye (SRB) and 3-(4,5-Dimethylthiazol-2-yl)-2,5-Diphenyltetrazolium Bromide (MTT) assays. Another test tried to assess if these conjugates were also dependent on the hENT-1 transporter as gemcitabine alone is. This experiment consisted of using an inhibitor of this transporter (nitrobenzylthionosine) when exposing BxPC3 to these conjugates and assessing the IC_50_ [20].

The results show that conjugates have higher inhibitory activity in PC-3 and MCF-7 cells than reference drugs (except for CPP6-2 in PC-3 cells). It is also demonstrated that CPP6-1 and 3 have higher activity, showing that Trp inclusion works best if it is in the *N*-terminal position, the middle of the conjugates, as predicted. The secondary test showed that in BxPC3 cells the preferred transportation of these conjugates was through the hENT-1 transporter, as there was a higher IC_50_ when the inhibitor was present [20].

This study concludes that conjugation of gemcitabine with CPP6-1 and 3 created a substantial increase of cell growth inhibition in PC-3 cells compared with gemcitabine alone, which, again, suggests that conjugation of this drug with CPP can be promising in cancer treatment, mainly in prostate cancer [20].

A different group also thought about using CPPs to improve the gemcitabine anticancer effect [22]. Five different CPPs were synthesized by adding tryptophan to a polyarginine chain, in different positions (scattered evenly or all in the middle and with different lengths). These CPPs were conjugated with gemcitabine and were exposed to adenocarcinoma human alveolar basal epithelial (A549) cells, where the cellular toxicity, uptake, and antitumor effects were assessed. The tryptophan increased uptake, with the higher uptake ones being the CPP conjugates with a higher number of amino acids (12), regardless of tryptophan placement. Conjugates with three and six tryptophans, as well as the one with tryptophan in the middle, increased cytotoxicity compared with gemcitabine alone (in concentrations of 15 and 25 µM). The higher uptake can be related to direct translocation of the conjugates into cells, which, combined with their capacity to evade the P-glycoprotein in the membrane (that causes multidrug resistance), causes high intracellular concentrations and, hence, high cytotoxicity. This proves that these tryptophan-arginine CPPs can improve intracellular delivery of gemcitabine and, consequently, its anticancer activity [22].

Besides CPPs, other types of peptides have already been conjugated with gemcitabine to positive results. For example, a superior metabolic gemcitabine molecule (GSG) was created through the conjugation of a gonadotropin-releasing hormone receptor (GnRH-R) ligand peptide with gemcitabine [23]. This peptide is an agonistic analog of the gonadotropin-releasing hormone and binds to GnRH-R, which is overexpressed in prostate cancer, producing excessive testosterone. A study of the mechanistic and metabolic capacities, as well as toxicity of this conjugate, was performed, using liquid chromatography coupled with tandem mass spectrometry (LC-MS/MS) techniques, to quantify intracellular levels of active and inactive gemcitabine, in vivo exposure to measure testosterone levels, and in vitro assays with castration-resistant prostate cancer (DU145) cells. Results show higher production of the active gemcitabine metabolite and lower production of the inactive, shifting the dynamic balance to the active metabolite and causing higher anticancer activity [23].

In terms of hematotoxicity, GSG is less toxic than gemcitabine alone, with no toxicity at all after daily exposure in mice. Besides being a strong agonist of the GnRH-R, GSG also suppresses testosterone, which is a newly discovered GSG anticancer mechanism. All of these findings, coupled with the fact that this conjugate is more stable than gemcitabine alone (prolonging its bioavailability) show that this conjugate can be of great help in prostate cancer treatment [23].

Another type of gemcitabine-peptide conjugate is 4-(Arg-Gly-Asp-Val-amino)-1-[3,3-difluoro-4-hydroxy-5-(hydroxylmethyl)oxo-lan-2-yl]pyrimidin-2-one, known as RGDV-gemcitabine [24]. Several in vitro and in vivo studies were carried out, with various kinds of cancer cells. The results revealed an increase in the half-life of RGDV conjugate compared to gemcitabine alone, along with a decrease in drug resistance and no marrow, kidney, or liver toxic effects. This conjugate is smaller than 100 nm, which allows it to escape macrophages, staying in circulation without being metabolized. Analysis of tumor and organ extracts showed a tumor-target effect of the RGDV-gemcitabine conjugate, being present in the tumor but not in the organs [24].

The previous study demonstrated how the RGDV-gemcitabine conjugate behaves in the organism. Another study from the same group set out to understand the possible effects and action pathways of this conjugate in tumor metastasis and growth [25]. Mice and A549-cells were the subjects of this study. For the same concentration of RGDV-gemcitabine and gemcitabine alone, only the conjugate inhibited invasion, migration, and adhesion of the A549-cells, as well as wound healing of the monolayer, which all depend on pseudopodia extension. Hence, the conjugate prevented pseudopodia extension, which is crucial for cancer metastasis. All this inhibition was selective for the cancer cells. This translated into the in vivo results, which revealed a dose-dependent inhibition of metastasis and growth of the tumor implanted in mice for the RGDV-gemcitabine only. The possible pathways of action could be related to downregulation of matrix metalloprotein 2 and 9, for the metastasis inhibition, and tumor necrosis factor and interleukin-8 downregulation in serum, for the growth inhibition [25]. Combining these studies, it is clear to see that RGDV-gemcitabine conjugate has an incredibly high potential for therapeutic use in lung cancer.

An important aspect of gemcitabine-peptide conjugates is the linkers that connect the two. A study related to this produced three gemcitabine D-Lys^6^-GnRH: two that were linked by a small carbamate bond (2G_1_ and 2G_2_) and one that was longer with two anime bonds and one ester bond (GSHG) [26]. According to the chemical properties, the first two were expected to be more stable than GSHG. The antiproliferative activity in prostate and cancer cells, stability in cell culture medium and blood, and pharmacokinetics in mice were tested. Stability was higher for 2G_1_ and 2G_2_, with GSHG disintegrating quickly and releasing the gemcitabine to the bloodstream, which agrees with what was expected. This also explains the highest cytotoxicity of GSHG. This conjugate has more difficulty in entering cells than 2G_1_ and 2G_2_ analogs, probably due to its lower binding affinity with the GnRH-R. However, once inside the cells, GSHG releases gemcitabine far more quickly than the other two. None of the conjugates led to the formation of the inactive gemcitabine metabolite, which is a plus compared with gemcitabine alone. The study concluded that, with these results, GSHG could be used for short-term administration, while 2G_1_ and 2G_2_ could be used for long-term administration. It also demonstrated a clear difference between the conjugates which have the same peptide, simply by changing the linker [26].

## 4. Combination of Gemcitabine with Repurposed Drugs

Another strategy that can be implemented to repurpose gemcitabine is by combining it with other non-cancer drugs. In a study from our group, gemcitabine and 5-fluorouracil (5-FU), which is another traditional anticancer drug, were combined with three repurposed drugs, itraconazole, verapamil, and tacrine to assess their joined effect on cancerous cells. The main objective was to predict the effect of these combinations in humans using in vitro and in silico techniques [27].

**Figure 3:**
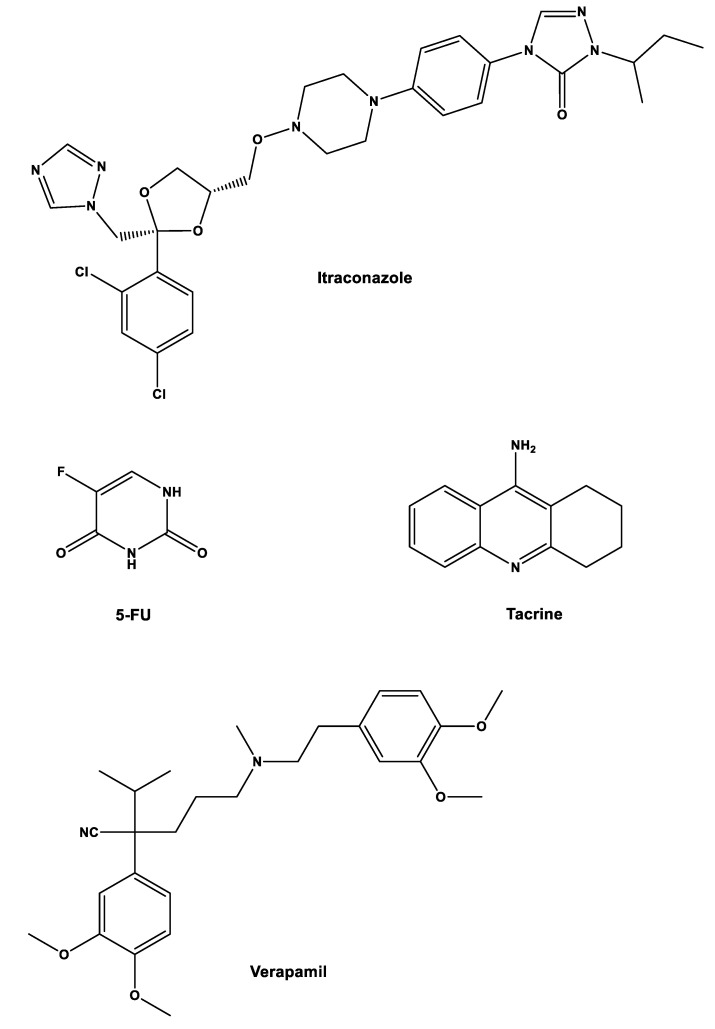
Structures of repurposed drugs used with gemcitabine for cancer projects.

Initially, six drug combinations were tested (one cancer drug with one repurposed drug, Figure 3) in three types of cells: non-small-cell lung cancer cell line A549; healthy and cancer human prostate cell lines (PNT2 and PC-3, respectively). Concentrations were 0.01–50 µM for gemcitabine, 0.05–100 µM for 5-FU, 1 µM for verapamil, 8.5 µM for itraconazole, and 0.24 µM for tacrine. The parameter assessed was cell growth inhibition with the MTT assay. The results showed that combinations with verapamil and tacrine had no effects on all types of cells, with the effects being the same as the ones with the anticancer drug alone, while combination with itraconazole improved the overall effect with 5-FU in all cell types and with gemcitabine in A549 cells. However, when looking at the effects of gemcitabine in PNT2 and PC-3 cells, the increase was only noticeable for smaller concentrations of this drug, matching with the control at higher concentrations of gemcitabine [27].

After these results were obtained, the effect of itraconazole concentration on drug combination responses was studied in A549 cells. This was achieved by two studies. The first combined a fixed concentration of gemcitabine (0.01 µM) and 5-FU (1 µM) with a range of concentrations between 0.07 and 4.25 µM of itraconazole. The second combined a range of gemcitabine (0.005–10 µM) and a range of 5-FU (maintained from the previous experiments) with a fixed concentration of itraconazole (2, 4, and 6 µM). The dose-response curves obtained for the first study showed that, at higher concentrations of itraconazole, the inhibition of cell growth for the gemcitabine-itraconazole combination is higher than for either drug alone, while for 5-FU-itraconazole, no improvement was obtained. In the second study, for both combinations, despite itraconazole not affecting the highest cell growth inhibition percentage, which is entirely dependent on the anticancer drugs, it had a great impact on the lowest cell growth inhibition percentage (from 0 to 13% and 33%), which seems to be dependent on itraconazole [27].

After the in vitro analysis, WinNonlin and STELLA software were used to develop two-compartment PK models that showed the effects of gemcitabine, 5-FU, itraconazole, and their combinations that were assessed previously together with literature pharmacokinetic (PK) values for humans. With these models, the area under the dose-response-time curve effect (AUC_effect_) values of the drug combinations in A549 cells were obtained. The results from these simulations showed that AUC_effect_ increased with higher concentrations of itraconazole combined with both anticancer drugs. Gemcitabine also had higher AUC_effect_ values since it has a higher dose and exposure time compared with 5-FU [27].

With these in situ models, the drug concentration in tissue and its impact on the % effect with time were also evaluated. It was shown that the % effect was higher in the gemcitabine-itraconazole combination (73% vs. 59% of cell growth inhibition) and it also maintained the high levels for a longer period (260 min vs. 70 min) before it started to drop abruptly. This type of relationship is important for dose regimen assessment [27].

Overall, this study demonstrated that itraconazole administrated with either gemcitabine or 5-FU is an interesting combination to potentially be used in the clinic and that PK models are of great importance and value in these types of works. It has also shown that gemcitabine can be combined with repurposed drugs to increase its effects as an anticancer drug without the need for chemical modifications [27].

## 5. PBPK in Drug Repurposing and Contributions to Personalized Medicine

As mentioned in the previous section, in silico studies can be of great use in repurposing investigation. The concept of physiologically based pharmacokinetic (PBPK) models first appeared in 1937, introduced by Teorell Torsten, the father of pharmacokinetics [28,29]. The mathematical framework is like the one used in the compartment model, but on PBPK models, besides the inclusion of a higher number of compartments, known anatomic and physiologic data are also included. This way, in a case example where pathology affects the physiology of an organ or tissue, the new physiology can also be taken into consideration. In PBPK models, contrarily to compartment models, relevant organs or tissues are realistically represented, which leads to more accurate drug distribution predictions [30,31].

**Figure 4:**
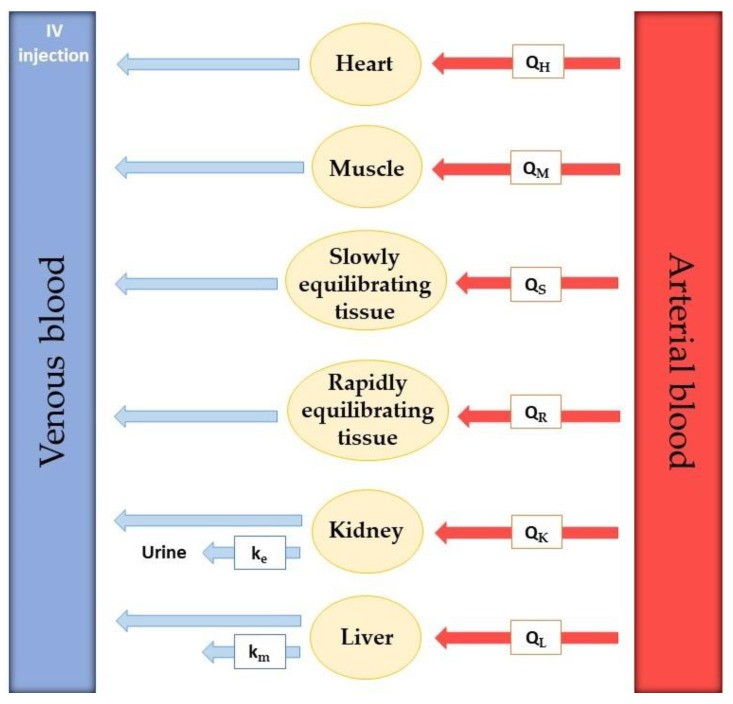
Representative example of a PBPK model for IV injection. Q’s represent the rate of blood perfusion in every compartment represented in the model. The compartment may be an organ or a group of tissues with a similar blood perfusion rate. Heart, Muscle, Slowly equilibrating tissue (SET), Rapidly equilibrating tissue (RET), Kidney, and Liver represent the different compartments of the model. k’s represent kinetic constants: k_e_ is the rate constant for drug excretion in the kidney and km is the rate constant for hepatic elimination. This model construction requires knowledge about the size or mass of each tissue compartment, which is experimentally determined.

Model development requires extensive experimental data collection. To realistically predict drug distribution throughout every part of the body, the rate of blood flow in every tissue is required. Besides that, real organ volume and tissue drug concentration must be described. The actual number of compartments included in the model is variable since tissues with no drug penetration are excluded. Usually, these are the brain, bones, and other parts of the central nervous system [30]. In Figure 4, an example of a PBPK model for drug perfusion after intravenous injection is shown. Relevant organs or tissues for drug absorption are shown separately, such as heart, muscle, kidney, and liver, while other tissues are grouped according to their ability to equilibrate with plasma. Tissues that equilibrate rapidly were included in the rapidly equilibrating tissue (RET) group and tissues that take longer to reach equilibrium were put in the slowly equilibrating tissue (SET) group. The rate of blood perfusion in every organ or tissue is known and represented by Q. Kidney and liver elimination rate constants are also included in the model (k_e_ and k_m_, respectively).

However, the model in Figure 4 is not completed. This is because there is no way for the drug to go from the injection IV to the tissues since all the arrows point to the left. The venous blood with the drug must go through the lungs and heart, where it becomes arterial blood, which then transports the drug to the tissues.

These PBPK models are very useful in personalized medicine since they consider different physiological data that allow the prediction of drugs’ pharmacokinetics in specific populations of patients. This allows one to study drug distribution according to age, health state, sex, and other factors, which subsequently allows for adjustment of treatment, such as dosage and kind of drug for that population, increasing therapeutic efficiency [32].

Unfortunately, much of the data required for PBPK model development are experimentally difficult to obtain, especially for human models. Therefore, this kind of approach is normally used to describe drug distribution in animal models, where experimental data are easier to obtain and comparison between different animal models is possible. By varying these animal models, insight into how physiologic and anatomic differences can affect drug distribution can be assessed, with possible extrapolation of data to a human model. Because of this, and despite PBPK models being useful, there are still many improvements that need to be made to fully put these techniques into practice.

Even though PBPK models are still in need of further development to be fully functional, other in silico methods are already being used in drug repurposing studies. For example, a study used mathematical models and experimental data to understand the effect of a sequential combination of gemcitabine and erlotinib in the cell cycle of two pancreatic cell lines (BxPC-3 and Capan-1) [33]. By using equations that mimic the evolution in the number of cells in each phase and age and inputting the probability of occurrence in each cell phase of block and delay (cytostatic effects) and death (cytotoxic), the authors developed the models that had the best fit with the experimental data (obtained from flow cytometry and time lapse). By doing this, they were able to explain exactly where each drug had altered the cell cycle [33].

For gemcitabine (with concentrations of 20, 40, and 120 nM), a delay and death in the S phase combined with a delay (without death) of G_1_ and a dose-dependent G_2_M block. This was congruent with the reduced DNA synthesis even for the lower concentration, with an incomplete recovery for higher concentrations (delay on S phase). These were the results obtained for BxPC-3 cells, with the same effects in Capan-1 cells, though it required a higher concentration. Using the effect models of the single drugs, simulations of sequential combined treatment were obtained (6 h incubation with the first drug, 18 h interval, and 48 h treatment with the second drug). Results showed that gemcitabine followed by erlotinib was synergetic in both cell lines, since first the gemcitabine-induced cytostatic effects were induced by arresting the S phase, and then this effect on the recovering cells was strengthened by erlotinib. This study clearly shows that gemcitabine can be used in combination with erlotinib with great effects in pancreatic cancer and that in silico models are of great value in understanding how drugs work [33].

## 6. Conclusions

In conclusion, it has been demonstrated in this perspective that great improvements can be made to gemcitabine that are sure to improve its anticancer properties. This can be accomplished either through chemical modifications, namely the addition of CPP, or through combination with non-anticancer drugs, such as itraconazole. With the aid of PBPK models, the effects of these modifications and the pharmacokinetics can be assessed in a specific subpopulation, allowing for personalized therapeutics. Despite this, these models need to continue being developed and improved, and more human data must be obtained for the full potential of PBPK to be unlocked.

## Data Availability

Not applicable.
